# Draft genome sequence of *Serratia proteamaculans* strain SK20 isolated from diseased rainbow trout (*Oncorhynchus mykiss*) in Türkiye

**DOI:** 10.1128/mra.00303-26

**Published:** 2026-05-06

**Authors:** Salih Kumru, Safak Kalindamar

**Affiliations:** 1 Faculty of Fisheries, Recep Tayyip Erdogan University 175650, Rize, Türkiye; 2 Faculty of Art and Science, Ordu University 187474, Ordu, Türkiye; California State University San Marcos, San Marcos, California, USA

**Keywords:** aquaculture, infectious disease, *Serratia proteamaculans*, trout

## Abstract

We report the draft genome sequence of *Serratia proteamaculans* strain SK20 isolated from diseased rainbow trout (*Oncorhynchus mykiss*) from an aquaculture facility in Türkiye. The genome is 5.38 Mb with 55% GC content and contains 5,012 predicted protein-coding genes.

## ANNOUNCEMENT

*Serratia* members are rod-shaped, Gram-negative, facultatively anaerobic bacteria widely distributed in diverse environments, including soil, water, plants, animals, and aquatic habitats (1–4). While *Serratia marcescens* is the most studied species due to its clinical importance, *Serratia proteamaculans* is recognized for its ecological versatility and opportunistic pathogenicity. This non-pigmented species has been associated with insect diseases, plant interactions, and food spoilage due to its proteolytic and lipolytic activities (5–9). Additionally, virulence factors such as protealysin and OmpX contribute to host interaction and invasion (10, 11). Despite its ecological importance, genomic data on fish-associated isolates remain limited.

*Serratia proteamaculans* strain SK20 was isolated in June 2011 from liver tissue obtained during routine diagnostic examination of a diseased rainbow trout (*Oncorhynchus mykiss*) from an aquaculture facility in Rize Province, Türkiye (41°01′17.5″N, 40°22′37.5″E). The liver tissue was aseptically processed by homogenization under sterile conditions, serially diluted (10⁻¹ to 10⁻⁶) in sterile phosphate-buffered saline, and plated on tryptic soy agar. Following incubation at 20°C for 24–48 h, a single colony was selected and purified by repeated streaking. The bacterium was grown aerobically in tryptic soy broth at 20°C with shaking at 150 rpm for 24–48 h. Cells were harvested by centrifugation, and genomic DNA (gDNA) was extracted using the Quick-DNA Fungal/Bacterial Miniprep Kit (Zymo Research) according to the manufacturer’s instructions without modification. gDNA quantity and quality were assessed using a Qubit 4.0 fluorometer (Invitrogen) and an Agilent 5400 Fragment Analyzer (Agilent). The sequencing library was prepared using the Nextera XT DNA Library Preparation Kit (Illumina) and sequenced on the Illumina NovaSeq 6000 platform, generating 4,684,275 paired-end reads (2 × 150 bp) (1.4 Gb). Raw read quality was evaluated using FastQC v.0.12.1 (12), and high-quality reads were *de novo* assembled using the CLC Genomics Workbench v.22.0.1 *de novo* assembler (CLC assembler module) with default parameters (similarity fraction: 0.99, word/bubble size: automatic). Genome annotation was performed using the NCBI Prokaryotic Genome Annotation Pipeline v.6.10 (13). Default parameters were used for all tools unless otherwise specified.

The assembly resulted in 20 contigs with an N50 value of 553.2 kb, a total genome size of 5,383,469 bp, and an average coverage of 256×. The draft genome contains 5,012 protein-coding genes, 73 tRNAs, and 1 rRNA operon, with approximately 55% GC content. Average nucleotide identity analysis revealed 99.6% similarity to *S. proteamaculans* CCUG 14510 (GenBank: WBKI00000000.1) (14, 15). Putative antimicrobial resistance genes were identified using the Resistance Gene Identifier v.6.0.5 with the CARD database v.4.0.1 (16). The SK20 genome contains putative genes encoding resistance to benzalkonium chloride, colistin, pulvomycin, tetracycline, cephalosporin, vancomycin, multidrug antibiotics, and beta-lactam antibiotics, based on strict hits ≥95% identity with a reference gene in the database. Furthermore, the genome contains putative genes encoding flagella, type I secretion system, type VI secretion system, and type IVa pili, which may contribute to several important processes, such as virulence, colonization, and bacterial competition.

## Data Availability

The final draft genome sequence of *Serratia proteamaculans* strain SK20 has been submitted to the National Center for Biotechnology Information (NCBI) genome database under the GenBank accession number JBSJDP000000000, BioProject number PRJNA1255297, BioSample number SAMN48139144, and SRA SRR37525866.
